# Arcuate fasciculus and pre-reading language development in children with prenatal alcohol exposure

**DOI:** 10.3389/fnins.2023.1174165

**Published:** 2023-06-02

**Authors:** Curtis Ostertag, Jess E. Reynolds, Preeti Kar, Deborah Dewey, W. Ben Gibbard, Christina Tortorelli, Catherine Lebel

**Affiliations:** ^1^Cumming School of Medicine, University of Calgary, Calgary, AB, Canada; ^2^Alberta Children’s Hospital Research Institute, University of Calgary, Calgary, AB, Canada; ^3^Hotchkiss Brain Institute, University of Calgary, Calgary, AB, Canada; ^4^Telethon Kids Institute, The University of Western Australia, Perth, WA, Australia; ^5^Department of Pediatrics, University of Calgary, Calgary, AB, Canada; ^6^Department of Community Health Sciences, University of Calgary, Calgary, AB, Canada; ^7^Department of Social Work, Mount Royal University, Calgary, AB, Canada; ^8^Department of Radiology, University of Calgary, Calgary, AB, Canada

**Keywords:** prenatal alcohol exposure, FASD, MRI, development, children, language, magnetic resonance imaging

## Abstract

**Introduction:**

Prenatal alcohol exposure (PAE) contributes to widespread neurodevelopmental challenges, including reading, and has been associated with altered white matter. Here, we aimed to investigate whether arcuate fasciculus (AF) development is associated with pre-reading language skills in young children with PAE.

**Methods:**

A total of 51 children with confirmed PAE (25 males; 5.6 ± 1.1 years) and 116 unexposed controls (57 males; 4.6 ± 1.2 years) underwent longitudinal diffusion tensor imaging (DTI), for a total of 111 scans from participants with PAE and 381 scans in the unexposed control group. We delineated the left and right AF and extracted mean fractional anisotropy (FA) and mean diffusivity (MD). Pre-reading language ability was assessed using age-standardized phonological processing (PP) and speeded naming (SN) scores of the NEPSY-II. Linear mixed effects models were run to determine the relationship between diffusion metrics and age, group, sex, and age-by-group interactions, with subject modeled as a random factor. A secondary mixed effect model analysis assessed the influence of white matter microstructure and PAE on pre-reading language ability using diffusion metric-by-age-by-group interactions, with 51 age- and sex-matched unexposed controls.

**Results:**

Phonological processing (PP) and SN scores were significantly lower in the PAE group (*p* < 0.001). In the right AF, there were significant age-by-group interactions for FA (*p* < 0.001) and MD (*p* = 0.0173). In the left AF, there was a nominally significant age-by-group interaction for MD that failed to survive correction (*p* = 0.0418). For the pre-reading analysis, a significant diffusion-by-age-by-group interaction was found for left FA (*p* = 0.0029) in predicting SN scores, and for the right FA (*p* = 0.00691) in predicting PP scores.

**Discussion:**

Children with PAE showed altered developmental trajectories for the AF, compared with unexposed controls. Children with PAE, regardless of age, showed altered brain-language relationships that resembled those seen in younger typically developing children. Our findings support the contention that altered developmental trajectories in the AF may be associated with functional outcomes in young children with PAE.

## 1. Introduction

Prenatal alcohol exposure (PAE) contributes to widespread neurodevelopmental and health challenges including cognitive and behavioral deficits, growth deficiencies, and/or craniofacial abnormalities (Cook et al., 2016; Mattson et al., 2019). Children who experience complex impacts of PAE may go on to be diagnosed with fetal alcohol spectrum disorder (FASD), which has an estimated prevalence of approximately 4% in North America (Cook et al., 2016; Flannigan et al., 2018; May et al., 2018; Popova et al., 2019).

Previous diffusion magnetic resonance imaging (dMRI) studies have reported extensive alterations to white matter microstructure in school-aged children, youth, and young adults with PAE (Lebel et al., 2008; Donald et al., 2015a; Ghazi Sherbaf et al., 2019), including lower fractional anisotropy (FA) and higher mean diffusivity (MD) compared to unexposed controls (Ghazi Sherbaf et al., 2019). These findings appear to be reversed in younger children, with higher FA and/or lower diffusivity in young children and infants with PAE compared to unexposed controls (Donald et al., 2015b; Kar et al., 2021). There are few longitudinal neuroimaging studies of PAE; however, they suggest that patterns of brain development are also altered in children with PAE, with slower maturation seen in young children (Kar et al., 2022) and faster development in older children (Treit et al., 2013). Longitudinal research is critical to capture the nuances of brain development during early childhood, which is a period of rapid brain growth (Lebel and Deoni, 2018). A better understanding of brain development at early ages may help identify sensitive periods for targeted interventions in children with PAE.

Prenatal alcohol exposure (PAE) negatively impacts cognitive and behavioral domains of functioning, including but not limited to general intelligence, motor skills, attention, executive function, learning, memory, and language (Mattson et al., 2019). Language and reading are areas of particular interest as deficits emerge during early childhood and can significantly influence social and educational outcomes. Worse receptive and expressive language abilities have been noted in children with FASD, and linked to problems with learning (Wyper and Rasmussen, 2011). Children and adolescents with PAE show deficits in reading comprehension and verbal fluency (O’Leary et al., 2013; Lindinger et al., 2022). A recent study showed comparable single word reading and phonological processing (PP) skills in adolescents with FASD compared to controls, but poorer reading comprehension, suggesting that even though the mechanics of reading may be intact at older ages in individuals with FASD, comprehension continues to be a problem (Lindinger et al., 2022).

In typically developing children, reading is supported by a network of left-lateralized brain regions in frontal, parietal, and temporal areas, including the Sylvian-parietal-temporal (SPT) area (Hickok and Poeppel, 2007; Buchsbaum and D’Esposito, 2008). These regions are connected by white matter tracts, which enable communication among regions and facilitate reading (Pugh et al., 2001; Vandermosten et al., 2012). White matter tracts classically associated with reading include the superior longitudinal fasciculus (SLF), inferior longitudinal fasciculus (ILF), inferior fronto-occipital fasciculus (IFOF), corona radiata, and uncinate fasciculus (UF) (Vandermosten et al., 2012; Lebel et al., 2019a; Meisler and Gabrieli, 2022). Another important region is the arcuate fasciculus (AF), a subdivision of the SLF, which is a white matter tract that plays a critical role in language processing, connecting the speech comprehension (Wernicke’s) area in the posterior temporal lobe to the speech production (Broca’s) area in the frontal lobe (Hickok and Poeppel, 2004). In conjunction with the SPT area, it maps sound-motor integration and supports phonological aspects of reading and speech (Vigneau et al., 2006), making it an interesting target of study in the context of PAE. In the AF of typically developing children, FA increases with age while MD decreases with age (Reynolds et al., 2019b). Notably, AF macrostructure is left lateralized in adults and typically developing children as young as 2 years of age (Lebel and Beaulieu, 2009; Qiu et al., 2011; Reynolds et al., 2019b), whereas functional and microstructural lateralization of the AF appear to develop more gradually across childhood (Reynolds et al., 2019b). FA, MD, and lateralization of the AF have all been linked to reading skills in school-aged children (Lebel and Beaulieu, 2009; Cheema et al., 2018). Similar relationships have also been observed in younger children, with AF microstructure linked to pre-reading language skills (Saygin et al., 2013; Reynolds et al., 2019b).

The neural correlates of reading and language difficulties in individuals with PAE are not well understood. One fMRI study found greater activation in the right precentral gyrus during PP tasks, as well as more rightward lateralization of FA in the ILF in children and adolescents with FASD compared to children with PAE but without FASD diagnoses or typically developing controls (Yu et al., 2022); however, they found no group differences in arcuate or SLF FA. Studies of the whole SLF (including the arcuate) report no microstructural differences (Kar et al., 2021) or lower FA compared to unexposed controls (Lebel et al., 2008). These varying results suggest that the AF requires further study to pinpoint microstructural abnormalities and to better understand how they may relate to reading difficulties in children with FASD. This is especially important to understand in early childhood as pre-reading skills develop rapidly and lay the foundation for later reading acquisition (Lonigan et al., 2000). Understanding the microstructural development of the AF and how this is associated with pre-reading skills in children with PAE will help in identifying the neurobiological correlates of reading difficulties in FASD and when they emerge.

The dynamic nature of brain maturation, especially during early childhood, makes longitudinal studies of white matter development critical in connecting brain alterations to functional outcomes in PAE. Early childhood is especially important to understand, as it is a time when many difficulties associated with PAE first become apparent, yet it remains largely unexplored in neuroimaging studies of PAE (Donald et al., 2015a). This study aimed to characterize development of AF microstructure in young children with PAE, and investigate its relationship to pre-reading language skills, using longitudinal MR imaging.

## 2. Materials and methods

### 2.1. Participants with PAE

**FIGURE 1:**
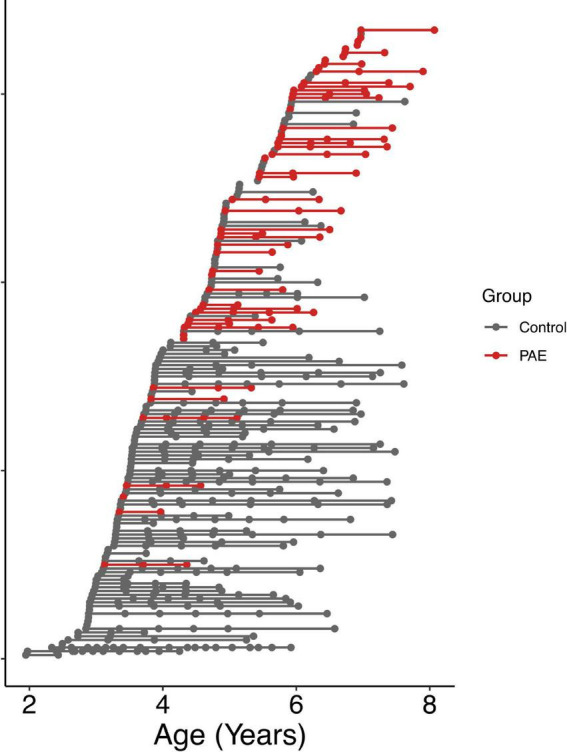
Age at MRI scans for all participants. Each row shows a different individual participant, ordered by age at first scan. Data points from the same participant are connected by a line. The PAE group is shown in red, and the unexposed control group is shown in gray.

A total of 57 children between 2 and 7 years of age with PAE were recruited through caregiver support groups, early intervention services, and the Ministry of Children’s Services in Alberta, Canada. Exclusion criteria were: birth before 34 weeks’ gestation, English not a primary language for the child, a history of head trauma, a diagnosis of autism spectrum disorder, cerebral palsy, epilepsy or any other medical or genetic disorder associated with serious motor or cognitive disability, and contraindications to MRI (e.g., metal implants, dental braces). Children with attention deficit hyperactivity disorder, learning disabilities, language delays, and/or mental health diagnoses were included, as these diagnoses are frequently comorbid with PAE. No participants had been diagnosed with FASD, as most clinics in Alberta do not assess children for FASD until age 7 years. Of the 57 children recruited, one was excluded for an incidental finding on MRI, 2 children did not feel comfortable participating in MRI scanning, and 3 children were excluded at analysis due to our inability to delineate the left and right arcuate. The final sample included in the analysis consists of 51 children (25 males/26 females; mean age for all scans: 5.65 ± 1.11 years) who provided a total of 111 diffusion tensor imaging (DTI) scans (2.2 ± 0.9 scans/participant); age at scan ranged from 3.13 to 8.07 years. The mean age at first scan was 5.3 ± 1.1 years. The distribution of number of scans per participant was as follows: 14 participants with 1 scan, 17 participants with 2 scans, 17 participants with 3 scans, and 3 participants with 4 scans (Figure 1). Other neuroimaging results from these children have been published previously (Kar et al., 2021, 2022).

All participants had PAE confirmed via medical, legal, or child welfare files, as well as interviews with biological and/or adoptive/foster families. A total of 33% (*n* = 17) of participants had confirmed PAE greater than or equal to the threshold indicated by the Canadian guidelines for diagnosing FASD (Cook et al., 2016): ≥ 7 drinks in 1 week and/or two or more binge episodes (≥ 4 drinks at one time) during pregnancy. A total of 67% (*n* = 34) had confirmed PAE of an unspecified amount.

### 2.2. Control participants

Typically developing participants were part of the Calgary Preschool MRI Study (Reynolds et al., 2020) and were recruited from Calgary and surrounding areas, as well as the Alberta Pregnancy Outcomes and Nutrition (APrON) study (Kaplan et al., 2014; Letourneau et al., 2022). Inclusion criteria were born > 36 weeks’ gestation, English as the primary language, no contraindications to MRI scans, and no history of developmental disorders or brain trauma. From this cohort, there were 381 high quality scans in 116 participants (3.3 ± 2.7 scans/participant) ranging from 1.95 to 7.63 years (mean age for all scans: 4.61 ± 1.20 years). The mean age at first scan was 4.0 ± 1.0 years. The distribution of numbers of scans per participant ranged from 1 to 20 and is as follows: 39 participants with 1 scan, 22 participants with 2 scans, 8 participants with 3 scans, 11 participants with 4 scans, 14 participants with 5 scans, 12 participants with 6 scans, 8 participants with 7 scans, 1 participant with 12 scans, and 1 participant with 20 scans (Figure 1). Unexposed control participants had confirmed absence of PAE based on prospective questionnaires and interviews completed with the mothers during pregnancy that asked directly about alcohol consumption.

Parent/guardian written informed consent and child verbal assent were obtained for each participant. The University of Calgary Conjoint Health Research Ethics Board (CHREB) approved this study (REB14-2266, REB13-020).

### 2.3. Language assessments

Participants ≥ 3 years completed the PP and speeded naming (SN) subtests of the NEPSY-II (Korkman et al., 2007) at each MRI scan timepoint. Phonological processing (PP) Standard scores and SN Combined Scaled Scores were used for analysis. The population means for standard scores are 10, with scores below the 26th percentile (score of 8), reflecting deficits (Korkman et al., 2007).

### 2.4. MRI image acquisition

All imaging was conducted using the same General Electric 3T MR750w system and 32-channel head coil at the Alberta Children’s Hospital, Calgary, Canada. Children were scanned either while awake and watching a movie of their choice or while sleeping without sedation. Foam padding was used to minimize head motion. Prior to scanning, parents were provided with detailed information of MRI procedures and were invited to an optional practice MRI session in an MRI mock scanner to familiarize the child with the scanning environment (Thieba et al., 2018). Whole-brain diffusion weighted images were acquired in 4:03 min using single shot spin echo echo-planar imaging sequence with: 1.6 mm × 1.6 mm × 2.2 mm resolution (resampled to 0.78 mm × 0.78 mm × 2.2 mm on scanner), TR = 6,750 ms; TE = 79 ms, 30 gradient encoding directions at *b* = 750 s/mm^2^, and 5 interleaved images without diffusion encoding at *b* = 0 s/mm^2^.

### 2.5. Data processing

Diffusion tensor imaging (DTI) data was visually inspected for quality, and volumes with artifacts or motion corruption were removed, per our previous methods (Walton et al., 2018; Reynolds et al., 2019a). All datasets included in the analysis had ≥ 18 high-quality volumes. Following corrupted volume removal, data was pipelined through ExploreDTI (V4.8.6) for correction for signal drift, Gibbs ringing (non-DWIs), subject motion, and eddy current distortions (Leemans et al., 2009).

Fiber tracking was performed using a deterministic streamline method in ExploreDTI. A minimum FA threshold of 0.20 was set to initiate and continue tracking, with angle threshold set to 30°. Using *a priori* location information on AF anatomy (Wakana et al., 2004; Lebel and Beaulieu, 2009), inclusion and exclusion regions of interest (ROI) were drawn on the left and right hemisphere separately, in each subject, per our previously published methods (Reynolds et al., 2019b). The number of streamlines, and mean values of FA and MD (mm^2^/s) were extracted for each tract. FA and MD mean values were calculated by computing an average of all voxels containing a portion of the tract, weighted by the number of streamlines intersecting with each voxel. Tracts with fewer than 10 streamlines were deemed unsuccessful and excluded from analysis. The left arcuate was successfully delineated in 111 of the 122 PAE datasets acquired (91%) and all 381 of the unexposed control datasets (100%). The mean number of streamlines for the left AF was 231 ± 144 in the PAE datasets and 295 ± 169 in the control datasets. The right arcuate could not be delineated in 31 of the 122 PAE datasets (25%) and 38 of the 381 unexposed control datasets (10%). The mean number of streamlines for the right AF was 151 ± 141 in the PAE datasets and 155 ± 122 in the control datasets.

### 2.6. Statistical analysis

RStudio version 1.4.1106 (R Core Team, 2020) and packages “lme4” (Bates et al., 2015), “lmerTest” (Kuznetsova et al., 2017) and “ggplot2” (Wickham, 2016) were used to carry out statistical analysis and plotting. Two-tailed *t*-tests were used to test group differences in pre-reading language scores (PP and SN), with a chi-square test used to test the distribution of scores below the < 26th percentile (Korkman et al., 2007) between each group. Linear mixed effects models were run to determine linear age-related changes in FA and MD for the left and right arcuate, as well as an asymmetry index [calculated as: (L-R)/(L + R)]. A positive asymmetry index for FA (higher FA in the left AF) and a negative asymmetry index for MD (lower FA in the left AF) indicate leftward asymmetry. Linear (y = age + sex + group + age*group + [1| Subject]) terms were modeled with age, sex, and age-group interactions as fixed factors and subject as a random factor. Restricted maximum likelihood was set to false. The main measure of interest was age-group interactions, which indicates different linear growth trajectories between groups. MD values were scaled by 1,000 to make them like other values for analysis. False discovery rate (FDR) was used to correct for 6 multiple comparisons of interaction terms (2 diffusion parameters for the right and left arcuate, as well as laterality index), with significance set at *q* < 0.05 (Benjamini and Hochberg, 1995).

The NEPSY-II has two different age bands, split at 5 years. The age-standardized scores allow comparisons of children younger and older than 5 years; however, the control and PAE groups differed in their mean age (see above). Therefore, we age-matched groups for analysis of language assessments to ensure balance is maintained across the age bands. A total of 51 typically developing children were matched to the PAE group by age at first scan and sex. This subset of controls had a total of 123 scans (2.4 ± 1.3 scans/participant) ranging from 3.17 to 7.61 years, with a mean age at first scan was 5.1 ± 1.0 years.

We used linear mixed effects models to investigate the association between diffusion metrics (FA/MD), age, and group on pre-reading language ability (PP/SN) (y = diffusion metric + age + sex + group + diffusion metric*age*group + [1| Subject]). PP/SN scores, diffusion metrics, and age were included as continuous variables within the model, while sex and group were categorical. The key outcome of interest was the relationship between diffusion metrics and pre-reading language ability at different ages. FDR correction was used to correct for 6 comparisons (2 diffusion parameters for the right and left arcuate, as well as laterality index) for both PP and SN models.

For plotting the pre-reading language analysis results, diffusion metrics were residualized for age and sex, and data points were split at age 5 (in accordance with the NEPSY-II age bands) to create 4 main group trajectories: PAE young, PAE old, unexposed young, and unexposed old.

## 3. Results

### 3.1. Participant characteristics

Participant characteristics are displayed in Table 1 (for the growth trajectories) and Table 2 (for the pre-reading language analysis subset). The mean PP score was 9.9 ± 3.0 in the PAE group and 12.0 ± 2.7 in the control group. The mean SN score was 10.5 ± 3.3 in the PAE group and 12.0 ± 2.9 in the unexposed control group. PP and SN scores were significantly lower in the PAE group compared to the unexposed controls (*p* < 0.001) (Table 2), though the mean scores in the PAE group are at the population mean (10). The PAE group had a significantly higher proportion of low PP scores (19 vs. 3% of datasets; *p* < 0.001) and low SN scores (19 vs. 7% of datasets; *p* = 0.016) than the controls.

**TABLE 1:** Participant characteristics for growth trajectories.

	PAE (*n* = 51; 111 datasets)	Control (*n* = 116; 381 datasets)	*p*
Mean age at first scan (years)	5.27 ± 1.06	3.97 ± 1.03	<0.001
Mean age across all scans (years)	5.65 ± 1.11	4.61 ± 1.20	<0.001
Age range (years)	3.13–8.07	1.95–7.63	
Mean number of scans per participant	2.18 ± 0.91	3.28 ± 2.71	0.005
Sex	25 M/26 F	57 M/59 F	0.828

PAE, prenatal alcohol exposure.

**TABLE 2:** Participant characteristics for the pre-reading language analysis subset.

	PAE (*n* = 51; 111 datasets)	Control (*n* = 51; 123 datasets)	*p*
Mean age at first scan (years)	5.27 ± 1.06	5.14 ± 0.99	0.54
Mean age across all scans (years)	5.65 ± 1.11	5.54 ± 1.04	0.42
Age range (years)	3.13–8.07	3.17–7.61	
Mean number of scans per participant	2.18 ± 0.91	2.35 ± 1.26	0.42
Sex	25 M/26 F	25 M/26 F	
Phonological processing std. score	9.94 ± 2.96	11.95 ± 2.66	< 0.001
Speeded naming combined scaled score	10.50 ± 3.26	11.96 ± 2.87	< 0.001

PAE, prenatal alcohol exposure.

### 3.2. Growth trajectories

**FIGURE 2:**
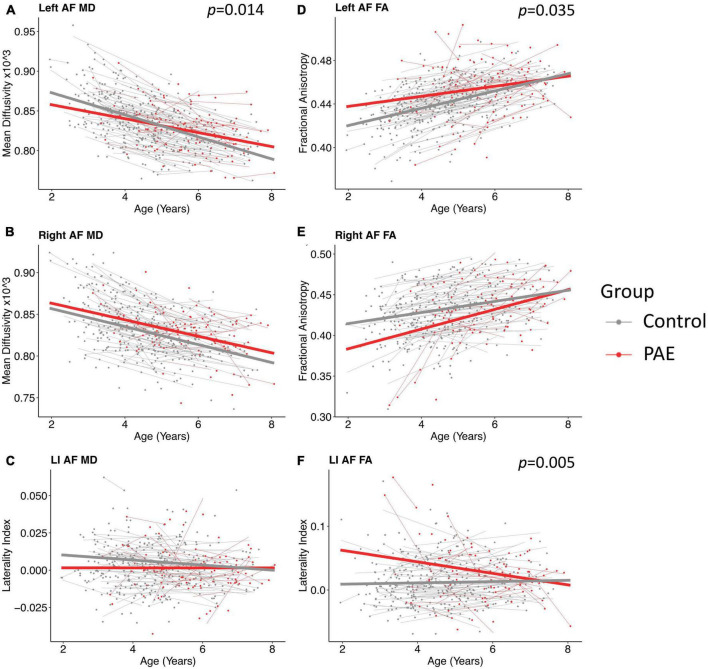
Linear growth trajectories for diffusion metrics in PAE and unexposed controls. Plots show individual data points as dots, with individual trajectories shown as thin lines, and the group trajectories displayed with thick lines. The PAE group is shown in red, while the unexposed control group is shown in gray. Plots **(A–C)** show the changes in mean diffusivity (MD) across ages. MD values in plots **(B,C)** are scaled up by 1,000. Plots **(D–F)** show the changes in fractional anisotropy across ages. Plots **(A)** and **(F)** show significant age-group interactions for MD in the left arcuate (*p* = 0.014; *q* = 0.043) and laterality index of FA (*p* = 0.005; *q* = 0.028), respectively. Plot **(D)** shows a nominally significant age-group interaction for FA in the left arcuate (*p* = 0.035), which failed to survive FDR correction (*q* = 0.07).

A significant age-group interaction was detected for MD in the left arcuate (*p* = 0.014, *q* = 0.043). There were no significant age-group interactions for MD in the right arcuate (*p* = 0.74) or for the laterality index (*p* = 0.29). For FA, there was a significant age-group interaction for laterality index (*p* = 0.005, *q* = 0.028). There was a nominally significant age-group interaction for FA in the left arcuate (*p* = 0.035) that failed to survive FDR correction (*q* = 0.07). The age-group interaction was not significant for FA in the right arcuate (*p* = 0.055). Growth trajectories are displayed in Figure 2, with model outputs in Tables 3, 4.

**TABLE 3:** Linear mixed effects model parameters for MD.

Mean diffusivity (x10^3^ mm^2^/s)	Parameter estimate (SE)	*t*	*p* (*q*)
**Left AF**			
Intercept	0.905 (0.004)	209.112	<2E-16***
Age	−0.014 (0.001)	−19.72	<2E-16***
Sex	−0.010 (0.004)	−2.792	0.006
Group	−0.025 (0.012)	−.063	0.040*
Age*group	0.005 (0.002)	2.46	0.014* (0.043)
**Right AF**			
Intercept	0.884 (0.005)	169.982	<2E-16***
Age	−0.011 (0.001)	−11.948	<2E-16***
Sex	−0.011 (0.004)	−2.773	0.006**
Group	0.005 (0.015)	0.316	0.752
Age*group	0.001 (0.003)	0.335	0.738
**Laterality index**			
Intercept	0.013 (0.003)	3.518	0.0005***
Age	−0.002 (0.001)	−2.813	0.005**
Sex	0.001 (0.002)	0.545	0.587
Group	−0.012 (0.009)	−1.324	0.186
Age*group	0.002 (0.002)	1.067	0.287

Results are shown for linear mixed models comparing age-related brain changes between children with PAE and unexposed controls. Significant differences in the main effects are shown as: *(*p* < 0.05), **(*p* < 0.01), ***(*p* < 0.001). Significant differences in the age-group interactions that survived multiple comparison (*q* < 0.05) are bolded, with the corresponding *q* value in parentheses. AF, arcuate fasciculus; SE, standard error.

**TABLE 4:** Linear mixed effects model parameters for FA.

Fractional anisotropy	Parameter estimate (SE)	*t*	*p*
**Left AF**			
Intercept	0.403 (0.003)	119.413	<2E-16***
Age	0.008 (0.001)	15.512	<2E-16***
Sex	0.002 (0.003)	0.764	0.446
Group	0.024 (0.009)	2.583	0.010*
Age*group	−0.003 (0.002)	−2.110	0.035*(0.07)
**Right AF**			
Intercept	0.397 (0.006)	71.182	<2E-16***
Age	0.007 (0.001)	7.407	1.1E-12***
Sex	0.007 (0.005)	1.575	0.117
Group	−0.042 (0.016)	−2.578	0.010*
Age*group	0.005 (0.003)	1.925	0.055
**Laterality index**			
Intercept	0.010 (0.007)	1.386	0.167
Age	0.001 (0.001)	0.812	0.418
Sex	−0.006 (0.005)	−1.040	0.300
Group	0.073 (0.021)	3.563	0.0004***
Age*group	−0.010 (0.004)	−2.840	0.005**(0.028)

Results are shown for linear mixed models comparing age-related brain changes between children with PAE and unexposed controls. Significant differences in the main effects are shown as: *(*p* < 0.05), **(*p* < 0.01), ***(*p* < 0.001). Significant differences in the age-group interactions that survived multiple comparison correction (*q* < 0.05) are bolded, with the corresponding *q* value in parentheses. Nominally significant age-group interactions that failed to survive multiple comparison correction are italicized, with the corresponding q value in parentheses. AF, arcuate fasciculus; SE, standard error.

Group main effects were significant in several models, including FA in the left arcuate (*p* = 0.010), the right arcuate (*p* = 0.010), and the laterality index (*p* = 0.0004); and MD in the left arcuate (*p* = 0.040). The PAE group showed higher overall FA in the left arcuate and higher (more leftward) FA laterality than controls, with lower FA in the right arcuate and lower MD in the left arcuate.

### 3.3. Pre-reading language models

**FIGURE 3:**
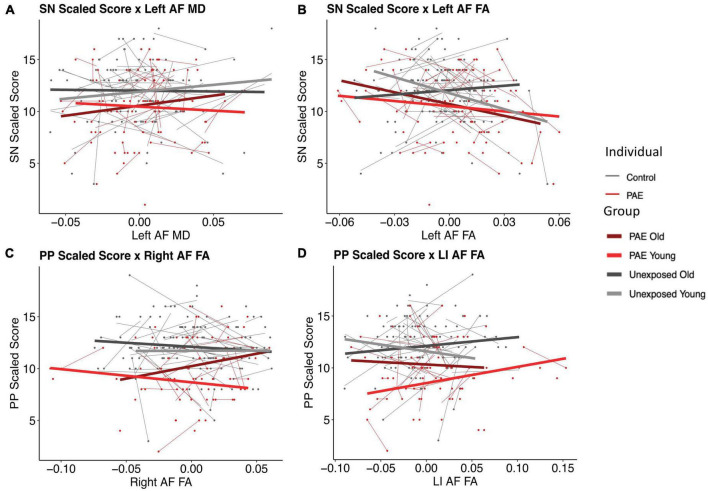
Plots of pre-reading scores by diffusion metrics. Diffusion metrics are shown as unstandardized residuals, controlled for age and sex. All plots shown display significant diffusion-age-group interactions. Individual data points are shown as dots, with individual trajectories fit by thin lines (PAE shown in red, unexposed controls shown in gray). Group fits are shown by the thicker lines, with the following color pattern: PAE old (dark red), PAE young (light red), unexposed old (dark gray), and unexposed young (light gray). Participants were split at age 5 to create the young and old plotting groups. Plots **(B)** and **(C)** show significant FA-age-group interactions for SN and the left arcuate (*p* = 0.003, *q* = 0.017) and PP and the right arcuate (*p* = 0.008, *q* = 0.0495), respectively. Plots **(A)** and **(D)** show nominally significant interactions that failed to survive FDR correction: an MD-age-group interaction for SN and the left arcuate (*p* = 0.031, *q* = 0.093) and a laterality index FA-age-group interaction for PP (*p* = 0.033, *q* = 0.098), respectively.

There were two significant three-way FA-age-group interactions detected: for PP and the right arcuate (*p* = 0.008, *q* = 0.0495) and for SN and the left arcuate (*p* = 0.003, *q* = 0.017) (Figure 3). There were two nominally significant interactions that failed to survive FDR correction: a laterality index FA-age-group interaction for PP (*p* = 0.033, *q* = 0.098) and an MD-age-group interaction for SN and the left arcuate (*p* = 0.031, *q* = 0.093) (Figure 3). All other three-way interactions were non-significant (see Tables 5, 6).

**TABLE 5:** Phonological processing model interaction outputs.

Phonological processing standard score	Parameter estimate (SE)	*t*	*p* (*q*)
**Left AF FA**			
FA*age*group	8.34 (14.54)	0.574	0.567
FA*group	−24.35 (78.03)	−0.312	0.755
FA*age	−10.12 (10.51)	−0.962	0.337
Age*group	−3.50 (6.55)	−0.535	0.593
Intercept	−14.51 (24.77)	−0.586	0.559
**Left AF MD**			
MD*age*group	1.86 (11.88)	0.157	0.875
MD*group	−24.93 (65.06)	−0.383	0.702
MD*age	0.51 (8.43)	0.061	0.952
Age*group	−1.42 (9.86)	−0.144	0.886
Intercept	10.91 (37.52)	0.291	0.772
**Right AF FA**			
FA*age*group	25.58 (9.58)	2.67	0.00825**(0.0495)
FA*group	−125.96 (52.42)	−2.403	0.017*
FA*age	−10.48 (7.02)	−1.493	0.138
Age*group	−10.55 (4.09)	−2.577	0.011**
Intercept	−13.15 (17.22)	−0.764	0.446
**Right AF MD**			
MD*age*group	−2.30 (11.70)	−0.197	0.844
MD*group	9.71 (64.34)	0.151	0.880
MD*age	3.36 (6.76)	0.498	0.620
Age*group	2.20 (9.71)	0.227	0.821
Intercept	24.73 (31.09)	0.795	0.428
**Laterality index FA**			
LI*age*group	−18.00 (8.36)	−2.154	0.0325*(0.0975)
LI*group	94.12 (46.53)	2.023	0.0446*
LI*age	7.45 (6.65)	1.121	0.264
Age*group	0.94 (0.42)	2.242	0.0261*
Intercept	10.22 (1.56)	6.544	6.97E-10***
**Laterality index MD**			
LI*age*group	6.87 (23.53)	0.292	0.771
LI*group	−56.45 (126.26)	−0.447	0.655
LI*age	−9.61 (13.97)	−0.7688	0.493
Age*group	0.29 (0.35)	0.848	0.398
Intercept	9.10 (1.29)	7.070	3.46E-11***

Results are shown for the linear mixed effects models that compared diffusion-age-group interactions in predicting PP scores. Only the interaction terms and the intercept are shown. Stars correspond to the following *p*-values: *(*p* < 0.05), **(*p* < 0.01), ***(*p* < 0.001). Significant three-way interactions that survived multiple comparison correction (*q* < 0.05) are bolded, with the corresponding *q* value in parentheses. Nominally significant three-way interactions that failed to survive multiple comparison correction are italicized, with the corresponding *q* value in parentheses. Significant two-way interactions and intercepts are starred, but not bolded, as these were not corrected for. AF, arcuate fasciculus; SE, standard error; FA, fractional anisotropy; MD, mean diffusivity; LI, laterality index.

**TABLE 6:** Speeded naming model interaction outputs.

Speeded naming combined scaled score	Parameter estimate (SE)	*t*	*p*
**Left AF FA**			
FA*age*group	−48.79 (16.16)	−3.019	0.0029**(0.0174)
FA*group	229.4 (86.73)	2.645	0.0088**
FA*age	43.62 (11.75)	3.714	0.00026***
Age*group	22.43 (7.28)	3.08	0.0023**
**Left AF MD**			
MD*age*group	29.40 (13.54)	2.172	0.031*(0.093)
MD*group	−141.75 (74.23)	−1.91	0.0576
MD*age	−24.24 (9.74)	−2.49	0.0139*
Age*group	−23.67 (11.23)	−2.11	0.0362*
**Right AF FA**			
FA*age*group	−10.49 (11.08)	−0.948	0.345
FA*group	63.82 (60.59)	1.053	0.294
FA*age	15.61 (8.17)	1.91	0.058
Age*group	5.12 (4.74)	1.081	0.281
**Right AF MD**			
MD*age*group	16.1 (13.38)	1.203	0.231
MD*group	−83.8 (73.9)	−1.133	0.259
MD*age	−3.7 (7.93)	−0.467	0.641
Age*group	−12.86 (11.11)	−1.158	0.248
**Laterality index FA**			
LI*age*group	−10.05 (9.75)	−1.031	0.3038
LI*group	37.17 (54.35)	0.685	0.494
LI*age	3.12 (7.79)	0.4	0.689
Age*group	0.77 (0.48)	1.595	0.112
**Laterality index MD**			
LI*age*group	15.03 (27.07)	0.555	0.579
LI*group	−65.72 (144.85)	−0.454	0.651
LI*age	−24.61 (16.14)	−1.525	0.130
Age*group	0.49 (0.39)	1.235	0.218

Results are shown for the linear mixed effects models that compared diffusion-age-group interactions in predicting SN scores. Only the interaction terms and the intercept are shown. Stars correspond to the following *p*-values: *(*p* < 0.05), **(*p* < 0.01), ***(*p* < 0.001). Significant three-way interactions that survived multiple comparison correction (*q* < 0.05) are bolded, with the corresponding *q* value in parentheses. Nominally significant three-way interactions that failed to survive multiple comparison correction are italicized, with the corresponding *q* value in parentheses. Significant two-way interactions and intercepts are starred, but not bolded, as these were not corrected for. AF, arcuate fasciculus; SE, standard error; FA, fractional anisotropy; MD, mean diffusivity; LI, laterality index.

## 4. Discussion

Here, we show altered developmental trajectories of AF microstructure in young children with PAE compared to unexposed controls. Our study also found reduced pre-reading language abilities in children with PAE compared to unexposed controls. Although both group means are in line with population norms (scores of ∼10), deficits (standard scores < 8) were more common in the PAE group.

The PAE group showed slower age-related decreases of MD in the AF than controls (see Tables 5, 6 for model interaction outputs and Supplementary Tables 1, 2 for model main effects). Initially, the PAE group had lower MD values than controls, but controls had steeper decreases of MD, leading to higher MD in the PAE group at the upper end of our age range. This aligns with our prior findings in the genu of the corpus callosum, the UF, the ILF, and the IFOF in the same participants (Kar et al., 2022). A similar pattern of slower development for FA was observed, although not significant, in the left AF. A recent study found no arcuate FA differences in adolescents with PAE-related facial dysmorphology (FASD/partial FASD) or heavy PAE non-FASD-diagnosed adolescents, compared to typically developing adolescents (Yu et al., 2022), perhaps suggesting these differences are more prominent at younger ages. These results are consistent with studies of other children at risk of reading disorders. For example, pre-reading children with a family history of dyslexia have lower FA and reduced leftward lateralization in the arcuate compared to children without a family history of dyslexia (Wang et al., 2017). Further, in both groups, the rate of FA development across the ILF, SLF, and arcuate positively correlated with reading development, suggesting that white matter development plays a role in both typical and atypical reading skill development (Wang et al., 2017). This may suggest children with PAE have similar neural correlates and developmental patterns to children at risk for other disorders associated with reading difficulties.

Lateralization of FA in the AF also showed significant group-by-age interactions. The PAE group began with strong leftward lateralization (higher FA in the left arcuate) and became more rightward lateralized over time; controls remained consistently left lateralized. The change in the PAE group was driven by steeper increases of FA in the right arcuate than in the left arcuate. Recent structural and functional MRI literature has demonstrated trends toward rightward lateralization of the language network in PAE and other disorders that impact reading. For example, adolescents with PAE show rightward laterality of FA in the ILF and greater activation of the right precentral gyrus during PP tasks than controls (Yu et al., 2022). Older children with dyslexia also show increased rightward laterality in white matter tracts associated with reading, including the IFOF and the SLF, with lateralization patterns related to individual differences in dyslexic children’s reading abilities (Zhao et al., 2016). Greater engagement of the right hemisphere in poor readers has been commonly noted across studies (Pugh et al., 2001; Shaywitz et al., 2002; Waldie et al., 2013), including in pre-readers at risk for reading difficulties (Ostertag et al., 2022), which suggests compensation mechanisms in which alternate pathways are recruited to process reading tasks in the context of dysfunctional left hemispheric reading networks. The altered trends in microstructural arcuate development found in this study may contribute to the development of atypical reading pathways and may underlie the reading impairments displayed in the PAE group.

Both pre-reading language scores were lower in the PAE group, and more participants with PAE had scores below the 26th percentile than controls (33 vs. 8%). Reading and language abilities are known to be reduced in older children and adolescents with PAE (O’Leary et al., 2013; Mattson et al., 2019; Lindinger et al., 2022). Here, we show that pre-reading language deficits are apparent in some children with PAE even before the emergence of fluent reading skills. This suggests an important link between brain and language development, similar to what has been previously observed in non-exposed children (Saygin et al., 2013; Wang et al., 2017; Reynolds et al., 2019b; De Vos et al., 2020).

The PAE group demonstrated an opposite relationship between SN scores and left arcuate FA compared to controls, such that better scores were associated with lower FA values. Interestingly, these trends mimic the younger age band of unexposed controls, suggesting that our findings may reflect brain immaturity. This is similar, although slightly less obvious, for the PP scores and right arcuate FA plot, such that both PAE groups (younger and older) showed trajectories of development of the arcuate similar to younger controls, with positive slopes, whereas the older controls show a negative slope. The faster development in the unexposed control group may represent a more dynamic period of establishing the expected brain-behavior relationships, which then are positively reflected in the older controls. The altered arcuate development in the PAE group could disrupt the organization of pre-reading language-microstructure relationships, thus reflecting the period of typical development where these relationships are theoretically weaker: younger ages. Considering the typical development of the AF where FA increases and MD decreases with age, with faster FA increases and MD decreases being related to better reading performance (Cheema et al., 2018), cohesiveness between white matter development and reading development is critical. Overall, these relationships suggest that the altered developmental trajectories in the arcuate could be associated with adverse functional pre-reading language outcomes.

## 5. Limitations and future directions

Brain alterations may depend on the timing, pattern, and amount of PAE, as well as exposures to other substances and experiences pre- or postnatally (Paintner et al., 2012; Lebel et al., 2019b; Hemingway et al., 2020; Flannigan et al., 2021). PAE was confirmed in all cases, however, precise information about dosage and timing is challenging to obtain retrospectively. Prospective studies that begin in the prenatal period and progress through childhood can capture more detail on PAE to compliment this work. Further, although pre-reading language skills have been linked to future reading outcomes (Wang et al., 2017), we cannot know which participants will go on to develop significant reading impairments. Future research should aim to pursue this issue longitudinally and identify early neural correlates of poor reading outcomes in children with PAE.

## 6. Conclusion

This was the first study to investigate microstructural development of the AF in young children with PAE. We found altered developmental trajectories for FA and MD in children with PAE when compared with unexposed controls. Pre-reading language skills were reduced in the PAE group, with scores low enough to be considered impaired significantly more prevalent in children with PAE. Relationships between pre-reading language skills and arcuate microstructure were altered in the PAE group, with children with PAE, regardless of age, displaying relationships that are more in line with a younger typically developing brain. This suggests that altered developmental trajectories are associated with adverse functional outcomes in young children with PAE.

## Data availability statement

The healthy control datasets presented in this study can be found in online repositories at https://osf.io/axz5r/. The data on participants with PAE is available upon request from the corresponding author and with appropriate ethics approval.

## Ethics statement

The studies involving human participants were reviewed and approved by the University of Calgary Conjoint Health Research Ethics Board. Written informed consent to participate in this study was provided by the participants’ legal guardian/next of kin.

## Author contributions

CO: conceptualization, methodology, data processing, analysis, investigation, writing the original draft, visualization, reviewing, and editing the draft. JR and PK: methodology, data collection and processing, investigation, reviewing, and editing the draft. DD: conceptualization, methodology, reviewing, editing the draft. WG: conceptualization, methodology, reviewing, editing the draft, and funding acquisition. CT: methodology, reviewing, and editing the draft. CL: conceptualization, methodology, writing the original draft, visualization, formal analysis, investigation, reviewing, editing the draft, project administration, funding acquisition, and overall project supervision. All authors contributed to the article and approved the submitted version.
