# Supplementary material for: Arcuate fasciculus and pre-reading language development in children with prenatal alcohol exposure
Source: Front Neurosci. 2023 Jun 2;17:1174165. doi: 10.3389/fnins.2023.1174165 (PMC10272404; doi:10.3389/fnins.2023.1174165)
Supplement: Supplementary file 1 [file Data_Sheet_1.PDF]

Supplementary Table S1: Phonological processing model main effect outputs.

**Phonological Processing Standard Score**	**Parameter Estimate (Std. Error)**	**t**	**p**
**Left AF FA**			
Intercept	-14.51 (24.77)	-0.586	0.559
FA	51.55 (55.41)	0.930	0.353
Age	5.11 (4.70)	1.086	0.279
Sex	-0.07 (0.49)	-0.145	0.885
Group	7.58 (35.08)	0.216	0.829
**Left AF MD**			
Intercept	10.91 (37.52)	0.291	0.772
MD	-2.81 (45.20)	-0.062	0.951
Age	0.15 (6.98)	0.022	0.983
Sex	-0.06 (0.49)	-0.119	0.906
Group	18.01 (54.23)	0.332	0.740
**Right AF FA**			
Intercept	-13.15 (17.22)	-0.764	0.446

FA	52.49 (40.21)	1.305	0.194
Age	4.94 (2.99)	1.649	0.101
Sex	-0.05 (0.48)	-0.108	0.915
Group	49.46	2.219	0.028 *
**Right AF MD**			
Intercept	24.73 (31.09)	0.795	0.428
MD	-18.97 (37.59)	-0.505	0.615
Age	-2.28 (5.59)	-0.408	0.684
Sex	-0.02 (0.49)	-0.040	0.968
Group	-11.69 (53.62)	-0.218	0.828
**Laterality Index FA**			
Intercept	10.22 (1.56)	6.544	6.97E-10 ***
LI FA	-36.04 (38.74)	-0.930	0.354
Age	0.27 (0.27)	0.996	0.321
Sex	-0.06 (0.48)	-0.124	0.902
Group	-7.26 (2.40)	-3.029	0.003 **

**Laterality Index MD**			
Intercept	9.10 (1.29)	7.070	3.46E-11 ***
LI MD	47.74 (77.62)	0.615	0.540
Age	0.49 (0.21)	2.315	0.022 *
Sex	-0.08 (0.48)	-0.163	0.871
Group	-3.69 (2.00)	-1.844	0.067

Note: Results are shown for the linear mixed effects models that compared diffusion-age-group interactions in predicting PP scores. Only the intercept and main effect terms are shown. Stars correspond to the following uncorrected *p* values: * ($p<0.05$), ** ($p<0.01$), ***($p<0.001$). The outputs for interaction terms are in Table 5.
Abbreviations: AF, arcuate fasciculus; SE, standard error; FA, fractional anisotropy; MD, mean diffusivity; LI, laterality index.

Supplementary Table S2: Speeded naming model main effect outputs.

Speeded Naming Combined Scaled Score	Parameter Estimate (Std. Error)	t	p
**Left AF FA**			
Intercept	115.6 (27.8)	4.157	4.77E-5 ***
FA	-233.76 (62.10)	-3.764	0.0002 ***
Age	-19.40 (5.26)	-3.687	0.0002 ***
Sex	0.56 (0.51)	1.102	0.273
Group	-107.48 (39.01)	-2.755	0.006 **

**Left AF MD**			
Intercept	-98.74 (43.34)	-2.278	0.024 *
MD	132.36 (52.24)	2.534	0.012 *
Age	20.18 (8.07)	2.501	0.013 *
Sex	0.64 (0.54)	1.194	0.234
Group	112.13 (61.83)	1.814	0.071
**Right AF FA**			
Intercept	53.1 (20.1)	2.648	0.009 **
FA	-98.77 (46.77)	-2.112	0.037 *
Age	-6.54 (3.49)	-1.875	0.063
Sex	0.58 (0.55)	1.038	0.302
Group	-32.73 (25.78)	-1.270	0.206
**Right AF MD**			
Intercept	-6.52 (36.59)	-0.178	0.859
MD	20.72 (44.26)	0.486	0.640
Age	3.21 (6.56)	0.490	0.625
Sex	0.69 (0.55)	1.255	0.213

Group	65.21 (61.63)	1.058	0.291
**Laterality Index FA**			
Intercept	11.26 (1.82)	6.183	4.54E-9
LI FA	-9.15 (45.45)	-0.201	0.841
Age	0.02 (0.32)	0.071	0.944
Sex	0.52 (0.54)	0.964	0.339
Group	-5.62 (2.78)	-2.022	0.045 *
**Laterality Index MD**			
Intercept	10.52 (1.48)	7.103	2.78E-11
LI MD	141.07 (89.83)	1.57	0.119
Age	0.16 (0.25)	0.65	0.517
Sex	0.65 (0.54)	1.21	0.230
Group	-4.34 (2.27)	-1.91	0.058

Note: Results are shown for the linear mixed effects models that compared diffusion-age-group interactions in predicting SN scores. Only the intercept and main effect terms are shown. Stars correspond to the following uncorrected p values: * ($p<0.05$), ** ($p<0.01$), ***($p<0.001$). The outputs for interaction terms are in Table 6.
Abbreviations: AF, arcuate fasciculus; SE, standard error; FA, fractional anisotropy; MD, mean diffusivity; LI, laterality index.
